# The Incidence and Correlation of Renal Pathologies Based on 14-Year Kidney Biopsy Material: A Retrospective Single-Centre Study in Poland

**DOI:** 10.3390/jcm15020495

**Published:** 2026-01-08

**Authors:** Krzysztof Benc, Ewa Tabaka, Wiktoria Pabian, Dominika Pisarek, Krzysztof Letachowicz, Tomasz Gołębiowski, Magdalena Kuriata-Kordek, Maciej Kanafa, Patryk Jerzak, Karolina Skalec, Piotr Donizy, Agnieszka Hałoń, Andrzej Konieczny, Mirosław Banasik

**Affiliations:** 1 Department of Nephrology, Transplantation Medicine and Internal Diseases Institute of Internal Diseases, Wroclaw Medical University, 50-556 Wroclaw, Poland; 2 Department of Clinical and Experimental Pathology, Wrocław Medical University, 50-556 Wrocław, Poland

**Keywords:** chronic kidney disease, large-core needle biopsy, glomerulonephritis

## Abstract

**Background:** In recent years, Poland has observed fluctuations in kidney biopsy frequency and shifts in diagnostic patterns. These trends likely reflect evolving clinical practice, diagnostic advancements, and changing disease epidemiology. This study aimed to analyse these changes, assess biopsy-based diagnoses across age groups, and examine sex-related variability. **Methods:** We conducted a single-centre, retrospective study at a university hospital in southwestern Poland, covering 2010–2024. Data from 1969 kidney biopsies were collected, within 1291 native kidney cases analysed after excluding transplant recipients. Diagnoses were correlated with patients’ age, sex, presence of diabetes, and temporal trends, and compared with previous studies. **Results:** Biopsy numbers increased over time, peaking in 2021 (154 procedures). Most were performed in patients aged 40–64 years (46.1%), followed by 18–39 years (39.1%) and ≥65 years (14.8%), with a rising proportion of elderly patients. Repeated biopsies occurred in 7.7% (second) and 0.6% (third biopsy). The most frequent diagnoses were IgAN (16.9%), FSGS (14.7%), and lupus nephritis (11.4%). In patients ≥65 years, amyloidosis (13.6%), FSGS (13.1%), vasculitis (13.1%), and membranous nephropathy (12%) predominated. The most marked sex-related difference involved lupus nephritis, accounting for 20.3% of diagnoses in women, who made up 82.3% of lupus nephritis cases. While most diseases showed male predominance, this was not evident for several, including IgAN and diabetic nephropathy. **Conclusions:** Given CKD’s underdiagnosis and frequent late detection in Poland, updated multicentre studies are needed to better recognise disease patterns and raise public awareness.

## 1. Introduction

Kidney diseases represent a major public health concern both in Poland [1] and worldwide [2,3]. According to the European Renal Association (ERA) Annual Registry, renal complications of diabetes (DM), hypertension (HT) and glomerular diseases are the main causes of chronic kidney disease (CKD), leading eventually to end-stage renal disease (ESRD), requiring one of the forms of renal replacement therapy (RRT). The incidence and distribution of specific renal pathologies have evolved over time, vitally influenced by several factors such as patients’ age, gender, environmental exposures, and socioeconomic trends [4,5]. These changes may be related to various causes, including the increased implementation of renal biopsy in diagnostics, especially in older populations [6], a greater awareness of lifestyle-related risk factors for kidney disease [7], and significant advances in diagnostic methods and pharmacological management [8,9,10].

Increasing knowledge on the role of comorbid conditions, such as obesity, HT, and DM, in both the development and subsequential progression of CKD has enhanced ability to identify and modify risk factors. This, in turn, allowed implementation of improved prevention strategies, delaying progression of CKD, and eventually reducing the incidence of ESRD and the subsequent need for RRT [11,12,13,14].

Lower Silesia has a population of approximately 2.9 million, with a notable percentage aged 65 and older. The prevalence of chronic kidney disease in Poland is around 13%, providing context for our findings.

Our study results indicate significant correlations between the observed disease proportions and regional health statistics, emphasizing the public health implications of kidney conditions.

Additionally, approximately 8% of the Polish population is diagnosed with diabetes, a known risk factor for kidney disease, highlighting the need for targeted public health initiatives in Lower Silesia.

These findings underscore the importance of early detection and intervention strategies for kidney disease in the region and address the reviewer’s request effectively.

Renal biopsy remains the gold standard for histopathological assessment of renal parenchymal diseases [15]. It enables precise diagnosis, disease staging, and prediction of the disease progression. When performed percutaneously, under ultrasound guidance, with appropriate patient’s preparation, renal biopsy is considered as a relatively safe procedure. However, thorough evaluation for potential contraindications is essential to minimise the risk of potential complications. These contraindications include coagulopathies, uncontrolled hypertension, active infections of the kidney or skin at the puncture site, and structural abnormalities such as a horseshoe kidney. The most severe bleeding may occur within the first 24 h after the procedure, typically within the initial 8 h [15,16]. The risk of complications strongly correlates with comorbidities such as cardiovascular disease, anaemia, thrombocytopenia, and tumours [16]. Other possible complications include microhaematuria (in almost all patients), macrohematuria (2–16%), arteriovenous fistula (0.5–10%), the need for nephrectomy (0.01–0.2%), blood transfusion requirement (2%), and rarely death (0.03–1.8%) [15].

While the epidemiology of kidney diseases in Poland has been widely studied [1,17,18,19,20,21], data focusing on the diagnosis distribution, based on the kidney biopsies, over the past several years, remain limited. Such analyses are crucial for improving diagnostic algorithms, prevention, and treatment of glomerulopathies.

The aim of this study is to retrospectively evaluate trends in histopathological diagnoses, based on kidney biopsies. The findings may provide a valuable reference for future epidemiological research and may support efforts to optimise the diagnostic and therapeutic approaches to adult patients with kidney disease.

## 2. Materials and Methods

Reports of all native kidney biopsies, performed in the single nephrology centre—Department of Nephrology, Transplantation Medicine and Internal Diseases Institute of Internal Diseases, Wroclaw Medical University, Wroclaw, in the years 2010–2024, were collected.

Subsequently, a retrospective analysis of medical records was performed, from which clinical and demographic data were obtained. The date of biopsy, patients’ age and sex, clinical diagnosis, and histopathological findings of the kidney biopsy were included into analysis. Patients with missing clinical and histopathological information, as well as those who could not be verified in the electronic patients’ database, were excluded from the study.

The data were saved in a database file. Statistical analyses were performed using Excel and R (version 4.5.0). Logistic regression analysis was also performed for binary dependent variables.

The study was approved by the Bioethics Committee of the Medical University of Wroclaw and complied with the Declaration of Helsinki.

## 3. Results

### 3.1. Patterns of Kidney Disease Presentation

All available data of 1291 adult patients, who underwent renal biopsy over the period 2010–2024, were analysed. Patients with the biopsy image non-diagnostic or inconclusive were also included.

Twenty-two following pathological diagnoses appeared at least once: IgA nephropathy (IgAN), focal segmental glomerular sclerosis (FSGS), lupus nephropathy (LN), membranous nephropathy (MN), ANCA related vasculitis, non-IgA mesangioproliferative glomerulonephritis (MesPGN), membranoproliferative glomerulonephritis (MPGN), non-specified chronic glomerulonephritis, amyloidosis (both AL and AA), diabetic nephropathy (DN), tubulointerstitial nephritis (TIN), minimal change disease (MCD), rapid progressive glomerulonephritis (RPGN), monoclonal gammopathy, hypertensive nephropathy, thrombotic microangiopathy (TMA), fibrillar glomerulopathy, necrotizing glomerulonephritis, leukemic infiltrates, thin basement membrane nephropathy, IgM nephropathy and acute intraepithelial nephritis.

The most frequent diagnosis in the entire study pool was IgAN with N = 218 (16.86%), predominantly in men N = 117 (53.67%). The second one was FSGS, accounting for N = 190 (14.69%) of all diagnoses, of which N = 123 (65%) were men. The third most frequent diagnosis was LN, accounting for 147 cases (11.37%), with women represented by N = 121 (88.2%). Diagnoses by gender, minimum, maximum and average age of patients are summarised in Table 1.

### 3.2. Analysis of Age-Related Trends in Glomerular Nephropathies

Patients were categorised into three age groups, as described below:

- Group I: ≥18–<40 years old
- Group II: ≥40–<65 years old
- Group III: ≥65 years old

The distribution of each age group was: 506 patients (39.1%) in age group I, 594 (46.1%) in age group II, and 191 (14.8%) was age group III at the time of biopsy.

**Figure 1:**
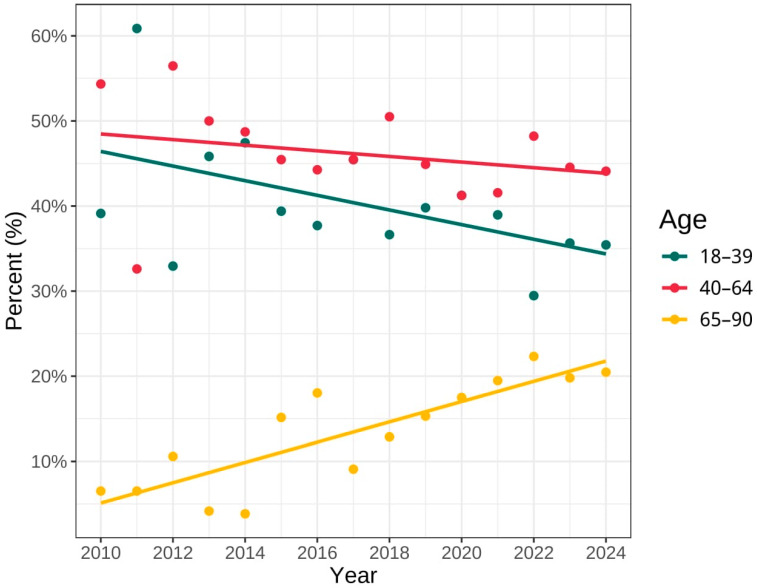
Percentage distribution of biopsies among different age groups.

Figure 1 illustrates a downward trend in the percentage share of age group I among patients undergoing biopsy procedures, over the years 2010–2024, with an estimated decrease of approximately 11%. Similarly, a declining trend was observed for age group II, with a decrease of around 4%. In age group III, there was an increase in the trend of number of the biopsy over the analysed period, amounting to 19%.

Table 2 provides a summary of the distribution of kidney diseases across the different age groups, along with the percentage of female and male patients within each group.

**Figure 2:**
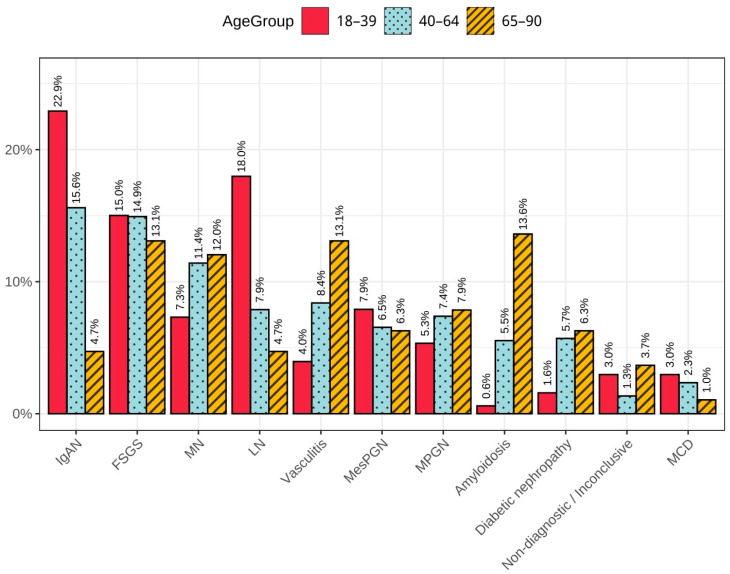
Distribution of selected diagnoses among all findings, stratified by age.

The analysis of the distribution of glomerular diseases showed that MesPGN occurred with similar frequency across all age groups, remaining at approximately 6–8% (Figure 2), with a slight predominance among younger individuals.

A similar stability was observed for FSGS, with the percentage of diagnoses around 15% in groups I and II, and slightly lower at 13% in group III. The percentage of TIN cases was evenly distributed within the range of 3–4.5%.

A clearly decreasing trend with age was observed for LN. Its frequency was approximately 18% in group I, dropping to 8% in group II, and further to around 4.8% in group III (Figure 2). The greatest increase in diagnoses occurred in the 20–29 and 35–39 age ranges.

A similar declining trend was noted for IgAN, decreasing from about 23% in group I, through 15% in group II, to just under 5% in group III. The highest proportion of IgAN diagnoses was in the 25–29 age range.

A comparable pattern, though with a much smaller percentage, was observed for MCD, with its frequency decreasing from 3% to 2.4%, and then to about 1%, respectively.

An opposite trend was observed in the case of ANCA-associated vasculitis—the proportion of cases increased from 4% in group I to 7% in group II, reaching approximately 13% in group III (Figure 2).

An upward trend with age was also characteristic of DN, with diagnosis rates of about 1.5% in group I, 5.5% in group II, and exceeding 6% in group III.

Amyloidosis showed a similar pattern: its frequency was around 1% among the youngest patients, 5% in the intermediate group, and about 13% in the oldest group. The peak incidence of amyloidosis was observed in the 55–79 age range, with a marked increase between ages 60 and 64.

For MPGN, an increase was noted across successive age groups, rising from 5.5% to just under 8%, while MN showed a rise from 8% to 11.5%.

### 3.3. Trend Analysis of Glomerular Nephropathy Distribution Based on Gender

**Figure 3:**
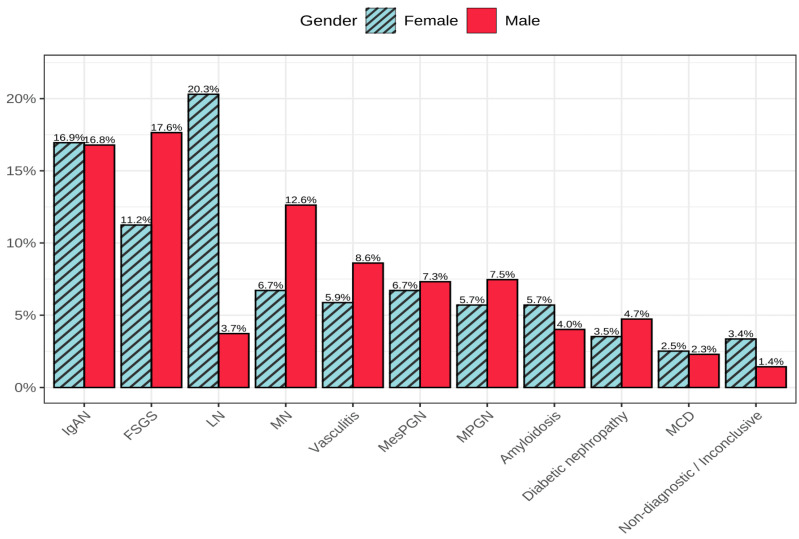
Distribution of selected diagnoses among all findings, stratified by sex.

The greatest gender disparity in the frequency of kidney pathologies was observed in LN, which was the most common diagnosis among women, accounting for as much as 20.3% (n = 121) of all diagnoses (Figure 3). In men, the same diagnosis was made in only 3.7% (n = 26) of cases.

Slightly more cases of amyloidosis were recorded in women, although this ratio changed with the patients’ age. In women from the second age group, amyloidosis occurred almost twice as often as in men, while in the third age group, the prevalence was similar for both genders.

IgAN was diagnosed in both genders with almost equal frequency—16.9% (n = 101) in women and 16.8% (n = 117) in men.

Diseases with similarly comparable percentages between genders included chronic glomerulonephritis, DN, TIN, and MCD.

In men, FSGS was the most frequently diagnosed disease, accounting for 17.6% (n = 123), while in women it was the third most common diagnosis at 11% (n = 67).

Cases of MN were nearly twice as common in men, representing 12.6% (n = 88), compared to 6.7% (n = 40) in women.

### 3.4. Epidemiological Trends in the Diagnosis of Glomerular Nephropathies from 2010 to 2024

The frequency of the biopsy procedure per year showed an increasing trend, with the highest result achieved in 2021 (N = 154), compared to 2020 (N = 80), 2022 (N = 112), 2023 (N = 101), 2024 (N = 127).

**Figure 4:**
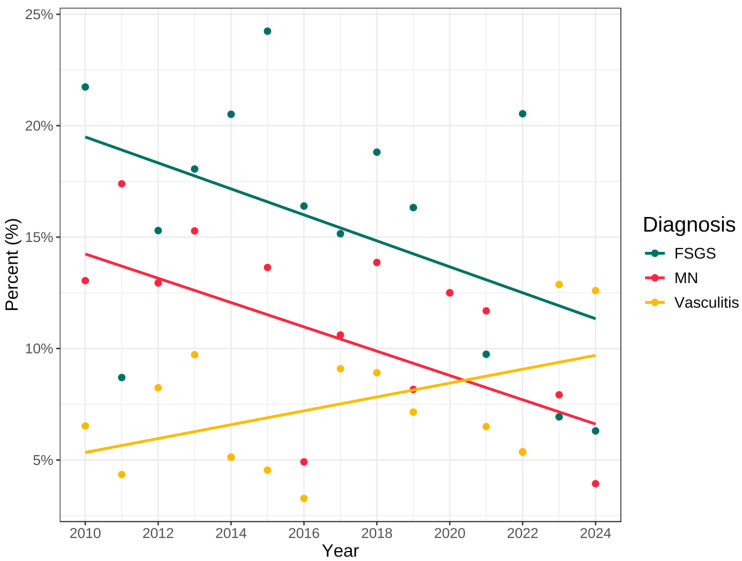
Yearly prevalence of selected diagnoses (FSGS, MN, vasculitis).

The analysis was limited to patients with native kidneys, who represented 65.67% (N = 1291) of the study population. Of these, 53.9% were male (N = 697), with a male-to-female ratio of 1.17. Among the entire pool of analysed cases, an inconclusive biopsy result, i.e., not allowing to establish the diagnosis with certainty, was described in 0.54% of patients (N = 7). A non-diagnostic picture, resulting mainly from an unrepresentative number of glomeruli taken for analysis, was presented by renal biopsy specimens of 1.62% of patients (N = 21). Analysis of the variability in disease diagnoses from 2010 to 2024 revealed a significant increase in the percentage of cases of vasculitis and amyloidosis. For vasculitis, the proportion was approximately 5% in 2010–2011, rising to about 12% in 2023–2024 (Figure 4). A similar trend was observed for amyloidosis, with its percentage increasing from around 4% at the beginning of the analysis period to 7% in 2024.

An opposite trend was seen in FSGS, where the frequency of diagnoses decreased from about 22% to 6% over the 14-year period. A declining trend was also noted for MN, dropping from approximately 15% to 5%.

No significant changes in diagnosis frequency were observed for the other disease entities during the analysed period.

### 3.5. Distribution of Kidney Diseases in Repeat Biopsies

**Figure 5:**
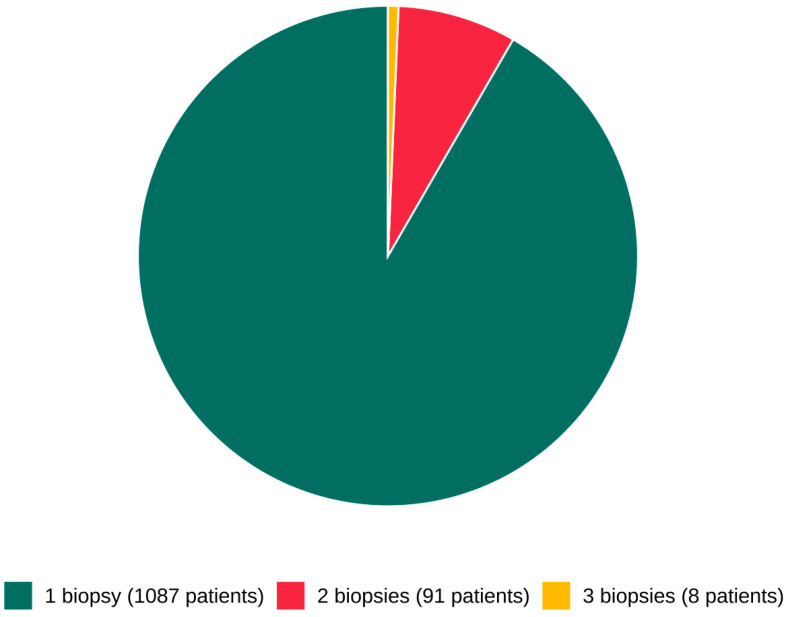
The number of biopsies performed on the patient.

The study population of 1291 patients with native kidneys were further divided according to the number of repeat biopsies performed. Patients who underwent a second biopsy accounted for 7.66% of the total group (n = 99) (Figure 5). A third biopsy was performed in 8.08% (n = 8) of those who had previously undergone a second biopsy, representing 0.62% of all patients included in the study. The most frequently diagnosed disease following the first biopsy was IgAN (15.7%). Among patients who underwent repeat biopsies, LN was the most common diagnosis—22.2% after the second and 37.5% after the third biopsy.

### 3.6. Distribution of Patients with Diabetes

Among the patients, 115 were diagnosed with DM. Of these, 61.7% (n = 71) were male. Type 2 DM was identified in 84.5% (n = 60) of the men and in 79.5% (n = 35) of the women, while the remaining individuals were diagnosed with type 1 DM.

An age-stratified analysis revealed that most patients were between 30 and 64 years of age. The distribution across age groups was as follows: Group II comprised 53.9% (n = 62), Group III accounted for 34.8% (n = 40), and Group I included 11.3% (n = 13) of the diabetic cohort.

The most frequent diagnosis based on kidney biopsy among diabetic patients was DN, observed in 44.3% (n = 51) of cases, with a predominance of type 2 DM (68.6%). Other common biopsy findings included vasculitis, diagnosed in 8.7% (n = 10) of patients, 90% of whom had type 2 DM; amyloidosis in 7.8% (n = 9), exclusively in patients with type 2 DM; and MN in another 7.8% (n = 9), also observed solely in the context of type 2 DM. Chronic glomerulonephritis was diagnosed in 7.0% (n = 8) of cases, with 87.5% occurring in patients with type 2 DM. Less frequent diagnoses included FSGS (5.2%), TIN (4.3%), IgAN (3.5%), MPGN (2.6%), monoclonal gammopathy and MCD, each accounting for 1.7%. Additionally, LN, hypertensive nephropathy, RPGN, and non-diagnostic biopsies were each identified in 0.9% of patients. In all these categories, type 2 DM was the predominant form.

Among diabetic patients, 93.1% (n = 91) had coexisting arterial hypertension, gastrointestinal disorders were present in 42.6% (n = 49), haematological diseases in 38.3% (n = 44), cardiovascular diseases in 28.7% (n = 33), and liver diseases in 27.0% (n = 31) of patients.

Other comorbid conditions, listed in descending order of frequency, included atherosclerosis (16.5%), diabetic retinopathy (13.0%), and nephrotic syndrome (9.3%).

## 4. Discussion

According to data from the Global Burden of Disease (GBD) report, there were nearly 674 million cases of CKD globally in 2021 [3]. Significantly, this number exceeds the number of people suffering from common chronic conditions such as DM, chronic respiratory disease, chronic cardiovascular disease, and osteoarthritis [22]. In the same year, CKD accounted for 1.5 million deaths worldwide, with mortality rate increasing with patients’ older age [3]. An analysis of GBD data showed that over the years of follow-up (1990–2021), the incidence of CKD increased significantly, reaching 21.5% [23]. In contrast, a large 2022 meta-analysis covering 218 countries indicates that 12.8% of the population may suffer from CKD in Eastern and Central Europe, which in Poland means potentially as many as 5 million people affected [2]. According to the Polish National Health Fund, in 2022, the standardised incidence of CKD was 345.75/100,000, which translates into 116,000 new diagnoses and 647,000 patients affected [1]. The significant discrepancy between the current prevalence registered in Poland and epidemiological estimates of large international studies may be due to the fact that the National Health Fund in Poland mainly reports cases of patients under permanent nephrological care and/or those qualified for RRT [20].

In our work, we have presented the distribution of specific GN and trends in its occurrence. We hope that the data we obtained will at least partially expand awareness of the epidemiology of kidney disease in Poland, although our study has its limitations.

An important aspect to mention is the rapid aging of the Polish population, which has been shown in many scientific studies, having a significant impact on the higher prevalence of CKD and subsequent mortality [1,24,25,26]. Our team conducted an analysis regarding the frequency distribution of conditions in the age group of the oldest patients (65 years and above).

Based on data from the Central Statistical Office in Poland, in 2023 the number of people >60 years of age amounted to 26.3% of the total Polish population, while in projections it is expected to reach almost 40% of the Polish population by 2060 [26]. The above data illustrate how significant is an issue of aging population in Poland. A large 2016 meta-analysis showed a linear upward trend in the prevalence of CKD in different age groups, ranging from 13.7% in the 30–40 age group to 27.9% in patients aged 70–80 [27]. Correspondingly, an increase in CKD mortality has also been shown, significantly correlating with older patient age [3,25].

Renal biopsy is an invasive diagnostic procedure that has been accepted as the gold standard for identifying kidney disorders and providing the opportunity for targeted therapy [3]. A worldwide consensus has now been adopted, stating that a patient’s older age is not a contraindication to perform biopsy [28,29]. Studies showed the absence of common serious adverse events [30,31]. In view of the above, we may accept the hypothesis that the proportion of elderly people in biopsy procedures will increase with the passing of years. The data we obtained confirm this assumption—over the 14 years of follow-up, the share of patients aged 65 and above increased, to eventually reach a 19% increase between 2010 and 2024. Relative to the results from the extensive Polish scientific work, the differences we noticed concerned the percentage share of each of the most common diagnoses.

The demonstrated results are coherent with the issue of understated occurrence of CKD worldwide, presented at the beginning of this discussion and may indicate evidence of many underdiagnosed cases, especially in the early stages of the disease. This leads to a situation in which patients obtain a diagnosis too late to introduce effective and targeted therapy.

Another noteworthy observation was the effect of the patient’s gender on the diagnosis. In our study, the male-to-female ratio was 1.17, indicating a slight male dominance. This is consistent with large scientific studies, invariably showing a higher incidence of kidney disease among men, as well as indicating a higher mortality rate compared to women in all age groups [3].

The scarcity of new scientific studies on the epidemiology of GN in Poland has partially limited our ability to analyse and compare the data we obtained. Based on the extensive nationwide report on the epidemiology of biopsy-confirmed renal disorders, created over the years 2009–2014, we can state with a high degree of certainty the similarities with our observations [18]. The outlined epidemiological ranking of diagnoses is analogous to other Polish data [17,18,21,32].

## 5. Conclusions

Chronic kidney disease remains a growing public health concern both in Poland and globally, particularly in the context of an aging population. Discrepancies between official registry data and epidemiological estimates indicate that the true prevalence of CKD is likely significantly underestimated. In the studied population of patients undergoing renal biopsy, the most frequently diagnosed forms of glomerulonephritis were IgAN, FSGS, LN, and MN. While the general distribution of diagnoses aligns with nationwide data in Poland, a higher proportion of LN and a lower proportion of MN were observed at the study centre.

Given the findings, efforts in the diagnosis of kidney diseases, particularly CKD—should emphasise earlier detection of glomerular pathology, ideally before progression to advanced stages. The notable number of cases classified as chronic glomerulonephritis suggests that many patients enter the diagnostic pathway at an already advanced stage of disease progression. This highlights the need for effective, accessible, and targeted screening programs, especially in high-risk populations, including elderly patients and those with comorbidities such as hypertension, diabetes, or persistent proteinuria/erythrocyturia.

It is essential to remind that the frequency of diagnoses presented in this study does not necessarily correlate with their clinical impact, particularly regarding conditions like end-stage renal disease (ESRD). While certain diagnoses may be reported less frequently, this does not imply that they have a lesser impact on patient health or outcomes. Some conditions, despite being diagnosed less often, can lead to significant health issues, including ESRD. Therefore, one should not interpret the data solely in terms of prevalence, as this may mislead the understanding of the clinical consequences of various renal pathologies.

Considering regional epidemiological variability and the challenges in obtaining comprehensive and standardised data, the creation of a national renal biopsy registry would provide significant benefits. Such a database would enable systematic collection and analysis of clinical, histopathological, and demographic information, facilitating real-time monitoring of disease trends, improving diagnostic accuracy, and informing health policy planning.

## Figures and Tables

**Table 1:** Diagnoses by min, max and average patient age.

Diagnosis	n ^1^	Min Age	Max Age	Mean Age	SD ^2^	Male (%)	Female (%)
IgAN	218	18	82	40	13.75	117 (53.67)	101 (46.33)
FSGS	190	19	90	46	16.15	123 (64.74)	67 (35.26)
LN	147	18	84	37	14.67	26 (17.69)	121 (82.31)
MN	128	20	83	50	15.00	40 (31.25)	88 (68.75)
Vasculitis	95	18	84	53	15.81	60 (63.16)	35 (36.84)
MesPGN	91	18	81	44	16.41	51 (56.04)	40 (43.96)
MPGN	86	18	78	48	16.55	52 (60.47)	34 (39.53)
Glomerulonephritis chronica	72	18	83	47	16.37	39 (54.17)	33 (45.83)
Amyloidosis	62	18	85	61	12.90	28 (45.16)	34 (54.84)
Diabetic nephropathy	54	20	80	55	14.06	33 (61.11)	21 (38.89)
TIN	52	21	78	47	15.32	30 (57.69)	22 (42.31)
MCD	31	18	72	40	15.57	16 (51.61)	15 (48.39)
RPGN	10	23	70	54	14.68	7 (70.00)	3 (30.00)
Monoclonal gammopathy	9	41	74	59	11.59	5 (55.56)	4 (44.44)
Hypertensive nephropathy	8	25	57	42	13.05	6 (75.00)	2 (25.00)
TMA	3	34	51	41	9.07	1 (33.33)	2 (66.67)
Arteriosclerosis	1	41	41	41	0.00	0 (0.00)	1 (100.00)
Fibrillary glomerulopathy	1	63	63	63	0.00	0 (0.00)	1 (100.00)
Necrotizing glomerulonephritis	1	18	18	18	0.00	1 (100.00)	0 (0.00)
Leukemic infiltration CLL/SLL	1	44	44	44	0.00	1 (100.00)	0 (0.00)
IgM nephropathy	1	24	24	24	0.00	1 (100.00)	0 (0.00)
Thin basement membrane nephropathy	1	26	26	26	0.00	1 (100.00)	0 (0.00)
Acute endocapillary glomerulonephritis	1	25	25	25	0.00	1 (100.00)	0 (0.00)
Non-diagnostic/Inconclusive	28	18	89	46	18.53	9 (32.14)	19 (67.86)

^1^ Number of cases, ^2^ Standard deviation.

**Table 2:** Distribution of kidney diseases by sex across different age groups.

Diagnosis	Total	18–39		40–64		65–90	
		Male	Female	Male	Female	Male	Female
IgAN	16.9%	25.2%	20.6%	14.2%	17.5%	4.0%	5.4%
FSGS	14.7%	19.7%	10.3%	16.9%	12.3%	15.2%	10.9%
LN	11.4%	5.5%	30.6%	3.2%	14.3%	1.0%	8.7%
MN	9.9%	9.4%	5.2%	15.4%	6.0%	11.1%	13.0%
Vasculitis	7.3%	3.9%	4.0%	9.9%	6.3%	16.2%	9.8%
MesPGN	7.0%	9.8%	6.0%	6.1%	7.1%	5.1%	7.6%
MPGN	6.7%	6.3%	4.4%	8.1%	6.3%	8.1%	7.6%
Glomerulonephritis chronica	5.6%	5.1%	5.2%	6.7%	4.4%	3.0%	9.8%
Amyloidosis	4.8%	0.4%	0.8%	3.8%	7.9%	14.1%	13.0%
Diabetic nephropathy	4.2%	1.2%	2.0%	6.7%	4.4%	7.1%	5.4%
TIN	4.0%	5.5%	2.4%	3.5%	5.6%	4.0%	2.2%
MCD	2.4%	2.8%	3.2%	2.0%	2.8%	2.0%	-
Non-diagnostic/Inconclusive	2.3%	1.6%	4.4%	0.9%	2.0%	3.0%	4.3%
RPGN	0.8%	0.8%	-	0.6%	0.8%	3.0%	1.1%
Monoclonal gammopathy	0.7%	-	-	0.6%	1.2%	3.0%	1.1%
Hypertensive nephropathy	0.6%	1.2%	0.4%	0.9%	0.4%	-	-
TMA	0.2%	-	0.8%	0.3%	-	-	-
Arteriosclerosis	0.1%	-	-	-	0.4%	-	-
Fibrillary glomerulopathy	0.1%	-	-	-	0.4%	-	-
Necrotizing glomerulonephritis	0.1%	0.4%	-	-	-	-	-
Leukemic infiltration CLL/SLL	0.1%	-	-	0.3%	-	-	-
IgM nephropathy	0.1%	0.4%	-	-	-	-	-
Thin basement membrane nephropathy	0.1%	0.4%	-	-	-	-	-
Acute endocapillary glomerulonephritis	0.1%	0.4%	-	-	-	-	-

## Data Availability

The original contributions presented in this study are included in the article. For further inquiries, please contact the corresponding author.

## Abbreviations

The following abbreviations are used in this manuscript:

CKD	Chronic kidney disease
ESRD	End-stage renal disease
IgAN	IgA nephropathy
FSGS	Focal segmental glomerulosclerosis
LN	Lupus nephritis
SLE	Systematic lupus erythematosus
MN	Membranous nephropathy
GN	Glomerulonephritis
MesPGN	Mesangial proliferative glomerulonephritis
MPGN	Membranoproliferative glomerulonephritis
TIN	Tubulointerstitial nephritis
MCD	Minimal change disease
RPGN	Rapidly progressive glomerulonephritis
TMA	Thrombotic microangiopathy
CLL	Chronic lymphocytic lymphoma
SLL	Small lymphocytic lymphoma
GFR	Glomerular filtration rate
PMP	Per million people
